# Toxicogenomics and Molecular Markers in Pollution

**DOI:** 10.3390/ijms23158280

**Published:** 2022-07-27

**Authors:** Maria Elena Crespo-Lopez

**Affiliations:** Laboratório de Farmacologia Molecular, Instituto de Ciências Biológicas, Universidade Federal do Pará, Belém 66075-110, PA, Brazil; maria.elena.crespo.lopez@gmail.com

Pollution is defined as the presence in or introduction of a substance into the environment that has harmful or poisonous effects. Anthropogenic activities are among the main causes of polluted environments and human exposure to pollutants. Powerful genomic tools have been developed in recent years to support the advancement of knowledge in many areas, including toxicology. In this Editorial, you can find a summary of the nine scientific papers published in the Special Issue “Toxicogenomics and Molecular Markers in Pollution” of the *International Journal of Molecular Sciences* (Table 1).

Interestingly, this topic has attracted the attention of researchers studying the effects of some of the pollutants that are of highest concern to public health at the moment: heavy metals, air pollution, nanomaterials and organic compounds such as the polycyclic aromatic hydrocarbon benzopyrene (BaP). In this Special Issue, readers will find new insights that have emerged from research ranging from in vitro studies to epidemiological analyses and pre-clinical studies using exciting models such as transgenic zebrafish. Shall we take a look?

Heavy metals are among the most dangerous pollutants for both the environment and human populations. In fact, the US Agency for Toxic Substances and Disease Registry currently ranks lead and mercury as the second and third substances with the highest potential to threaten human health [1]. This list is revised periodically based on frequency of occurrence, toxicity and the potential for human exposure. The fascinating study of Lu et al. [2] used the CRISPR/Cas9 system to create an ABCC4−/− mutant zebrafish line to analyze the role of this transporter in lead exposure. ABCC4 is an important transporter in the cellular efflux of many drugs and organic compounds, such as organochlorine pesticides. However, its role in lead detoxification is not well understood. Lead exposure induced ABCC4 expression during development, supporting its protective role. These authors demonstrated that ABCC4 can contribute to lead clearance, improving cellular survival and, consequently, reducing the mortality rate of animals. In addition, a new transgenic line (ABCC4+/−) was also developed, which allowed them to find that a new GSH-dependent mechanism involved in ABCC4-mediated lead transport in zebrafish

The zebrafish model has also been used in the study by Glazer and Brennan [3] to analyze the deleterious consequences for early development of exposure to low doses of methylmercury, the most toxic form of mercury. The main target organ of methylmercury is the Central Nervous System (CNS) [4,5], and the mechanisms underlying the damage caused by the metal include oxidative stress, excitotoxicity, calcium imbalance, DNA damage, neuroinflammation, altered neurotransmission, impaired neurogenesis and cell death, as reviewed by Novo et al. [6]. In addition to describing some of the outbreaks of mercury intoxication in human populations, this review also draws attention to the importance of furthering our understanding of the effects of low-dose exposure to the metal [6]. That is precisely what Glazer and Brennan [3] did in their study, finding that very low concentrations of methylmercury (as low as 5 nM) caused anxiety and an impaired stress response in both adults and developing animals. These symptoms have been previously detected in other models exposed to high doses of the metal, as well as other signs related to emotionality such as depression and insomnia [7]. However, the most interesting and novel result was the altered gene expression profile, which was associated with behavioral alterations, demonstrating the involvement of the dopaminergic system and the hypothalamic–pituitary–interrenal (HPI) axis in both stages of life (during development and in adulthood) exposed to low doses of MeHg [3]. This is important considering that all species of mercury can form deposits in the hypothalamus [7] and also because the HPI axis represents an example of the crosstalk between the brain and the periphery.

The crosstalk between the CNS and the periphery is precisely the basis for the development of peripheral biomarkers that support the early diagnosis of mercury-induced neurotoxicity [8]. Arrifano et al. [9] analyzed for the first time the role of extracellular vesicles (EVs) in mercury intoxication, revealing that the reduction in the number of exosomes could be a new mechanism associated with metal-induced neurotoxicity and that plasmatic EVs could represent a source of future biomarkers.

The analysis of molecular markers in blood has also been used by Honkova et al. [10] to study the consequences of air pollution on police officers working outdoors in different cities of the Czech Republic. Interestingly, genome-wide DNA methylation profiles demonstrated 13,643 CpG loci differentially methylated between groups based on air pollution type in each city. Alterations related to diabetes mellitus (KCNQ1), the dopaminergic system of the brain and neurodegenerative diseases (NR4A2) and respiratory diseases (PTPRN2), among others, were detected.

Albano et al. [11] have reviewed respiratory diseases caused by air pollution with a special focus on the role of epithelial cells. In addition, this interesting review provides a summary of new models (3D air–liquid interface cultures, organotypic cultures and lung-on-a-chip, among others) and tools (exposome, microRNAs and omics technologies) for the study of respiratory diseases related to pollution. In vitro/ex vivo cell models of human bronchial epithelial cells have already demonstrated the molecular and cellular mechanisms of pollutants such the genotoxicity of flame retardants [11].

Genotoxicity is the damage caused to DNA that can lead to loss of efficient control of the cell cycle, causing carcinogenic and teratogenic processes. Therefore, it is one of the most worrying long-term consequences of human exposure to pollutants. Some of these contaminants can cause these types of deleterious consequences, even with low levels of exposure, as is the case with some metals such as mercury [12]. The elegant work of Kreuzer et al. [13] reveals an additional aspect of genotoxic substances: a specific transcriptomic signature in blood cells. By comparing the effects of genotoxic compounds such as BaP in two in vitro models (liver and blood cell lines), these authors demonstrated that, unlike the cell line of liver origin, the lymphoblastoid cell line shows altered gene expression profiles specific for each genotoxin. Interestingly, these results were confirmed by bioinformatics, supporting the detection of these transcriptomic signatures as potential peripheral biomarkers.

**Table 1:** Summary of the nine articles included in the Special Issue “Toxicogenomics and Molecular Markers in Pollution” of the *International Journal of Molecular Sciences*.

Authors	Title	Type	DOI
Lu et al. [2]	Generation of Knockout and Transgenic Zebrafish to Characterize Abcc4 Functions in Detoxification and Efflux of Lead	Research Article	10.3390/ijms22042054
Glazer et al. [3]	Developmental Exposure to Low Concentrations of Methylmercury Causes Increase in Anxiety-Related Behaviour and Locomotor Impairments in Zebrafish	Research article	10.3390/ijms222010961
Novo et al. [6]	Cellular and Molecular Mechanisms Mediating Methylmercury Neurotoxicity and Neuroinflammation	Review	10.3390/ijms22063101
Luz et al. [7]	Methylmercury plus Ethanol Exposure: How Much Does This Combination Affect Emotionality?	Review	10.3390/ijms222313131
Arrifano et al. [9]	Contributing to Understand the Crosstalk between Brain and Periphery in Methylmercury Intoxication: Neurotoxicity and Extracellular Vesicles	Research article	10.3390/ijms221910855
Honkova et al. [10]	Genome-Wide DNA Methylation in Policemen Working in Cities Differing by Major Sources of Air Pollution	Research article	10.3390/ijms23031666
Albano et al. [11]	Impact of Air Pollution in Airway Diseases: Role of the Epithelial Cells (Cell Models and Biomarkers)	Review	10.3390/ijms23052799
Kreuzer et al. [13]	Comparative Analysis of Transcriptional Responses to Genotoxic and Non-Genotoxic Agents in the Blood Cell Model TK6 and the Liver Model HepaRG	Research article	10.3390/ijms23073420
Jiang et al. [14]	Dependence of Graphene Oxide (GO) Toxicity on Oxidation Level, Elemental Composition, and Size	Research article	10.3390/ijms221910578

To complete the Special Issue, Jiang et al. [14] used an innovative approach in toxicogenomics to analyze graphene oxide (GO), an important graphene-based material used in nanotechnology. These authors performed a three-dimensional high-throughput toxicogenomic-based analysis (exposure time, specific biomarker and expression alteration magnitude) employing a GFP-fused yeast reporter library to rapidly and effectively assess GO toxicity. Interestingly, the results showed that GO toxicity depends on particle size and composition and that UV-treatment of GO increases its toxicity, especially for proteins. Future studies may confirm that internalization of GO sheets plays a key role in its toxicity.
